# Posterior chamber collagen copolymer phakic intraocular lens with a central hole for moderate-to-high myopia: First experience in China

**DOI:** 10.1097/MD.0000000000004641

**Published:** 2016-09-09

**Authors:** Xinfang Cao, Weiliang Wu, Yang Wang, Chen Xie, Jianping Tong, Ye Shen

**Affiliations:** a Department of Ophthalmology, the First Affiliated Hospital, College of Medicine, Zhejiang University, Hangzhou, P.R. China.; b Department of Orthopedics, Children's Hospital, College of Medicine, Zhejiang University, Hangzhou, P.R. China.

**Keywords:** central hole, Chinese, implantable collamer lens, myopia, phakic intraocular lens

## Abstract

The purpose of this article is to evaluate the clinical outcomes of a posterior chamber phakic intraocular lens (pIOL) (Visian Implantable Collamer Lens V4c) for the correction of moderate to high myopia in Chinese eyes.

The article is designed as a retrospective case series.

This study included the first consecutive eyes that had implantation of a new pIOL design with a central hole, at our department by the same surgeon. The safety, efficacy, predictability, stability, and adverse events of the surgery were evaluated over 6 months.

The study enrolled 63 eyes (32 patients). The mean spherical equivalent decreased from −12.81 ± 3.11 diopters (D) preoperatively to −0.05 ± 0.27 D 6 months postoperatively; 96.8% of eyes were within ±0.50 D of the target and 100% of eyes were within ±1.00 D. All eyes had a decimal uncorrected distance visual acuity of 0.5 (20/40) or better at every follow-up visit. The safety and efficacy indices were 1.42 ± 0.34 and 1.11 ± 0.19, respectively. Postoperatively, the intraocular pressure (IOP) remained stable over time. No significant rises in IOP (including pupillary block) and no secondary cataract were found. After 6 months, the mean vault was 505.2 ± 258.9 μm (range 120–990 μm), and the mean endothelial cell loss was 2.0%.

Implantation of the pIOL was safe, effective, predictable, and stable in the correction of moderate-to-high myopia in Han Chinese patients, even without peripheral iridectomy.

## 1 Introduction

The Visian Implantable Collamer Lens (ICL; STAAR Surgical Co., Switzerland) is a foldable phakic intraocular lens (pIOL) designed to be placed in the posterior chamber behind the iris with the haptic zone resting on the ciliary sulcus.^[1]^ It is currently the most widely used posterior chamber pIOL (PC-pIOL) worldwide for surgical correction of moderate-to-high ametropia. The implantation of ICL is a removable procedure that provides highly predictable and stable results while preserving accommodation.^[2]^ In order to improve visual quality and reduce the incidence of complications, mainly pupillary block and lens opacities, the initial ICL V1 model has evolved to the ICL V4c model by introducing several changes in the design.^[3]^ Shimizu,^[4–6]^ in cooperation with STAAR Surgical Co, developed the Visian ICL V4c with a central artificial hole in the center of the ICL optic. This new development improves aqueous humor circulation in the eye and eliminates the need for a preoperative laser iridotomy or intraoperative iridectomy.^[6–9]^

A few studies have focused on this model and reported good refractive outcomes in terms of predictability, safety, efficacy, and stability through the first months after surgery.^[2,7,8]^ However, most of the studies were performed in the eyes of white patients. There is a lack of data in the literature regarding the clinical outcomes of Han Chinese eyes with high and super high myopia implanted with ICL V4c. Since October 2014, this new pIOL model with a central hole was approved to be used in China. We, from November 2014, began to carry out the implantation of the Visian ICL V4c for the treatment of myopia at our department. To fill in this gap, we report the first clinical and refractive outcomes of Hole ICL implantation in Chinese eyes with moderate-to-high myopia.

## 2 Patients and methods

This study included the first consecutive eyes having implantation of myopic V4c Visian ICL pIOLs for the correction of moderate-to-high myopia at the Department of Ophthalmology, First Affiliated Hospital, College of Medicine, Zhejiang University, China. All patients provided written informed consent after the nature, and possible consequences of the study were explained fully in accordance with the Declaration of Helsinki. The study was approved by the ethics committee of the First Affiliated Hospital.

The inclusion criteria for pIOL implantation were ages between 21 years and 45 years, stable refraction with a myopic refractive error in the range correctable with the V4c pIOL (from −0.50 to −18.00 diopters [D]), a clear central cornea. Exclusion criteria were keratoconus, previous refractive surgery, glaucoma, cataract, uveitis, history of retinal detachment, anterior chamber depth (ACD) <2.8 mm, endothelial cell density (ECD) <2000 cell/mm^2^.

### 2.1 Preoperative examination

Before surgery, patients had a complete ophthalmologic examination including uncorrected distance visual acuity (UDVA), corrected distance visual acuity (CDVA), manifest and cycloplegic refractions, keratometry, corneal topography, pachymetry using scanning-slit corneal topography (Orbscan II, Bausch & Lomb, USA), ECD, A-scan ultrasonography, slitlamp microscopy, tonometry, and dilated indirect fundoscopy.

### 2.2 Intraocular lens

ICL V4c is available in 4 overall lengths as follows: 12.1 mm, 12.6 mm, 13.2 mm, and 13.7 mm. It is designed to correct myopia in a power range from −0.50 to −18.00 D.^[2]^ The postoperative targeted refraction was emmetropia in all cases. Power calculation for the pIOL was performed using the software provided by the pIOL manufacturer and a modified vertex formula.^[1,9]^ The pIOL diameter was individually determined based on the horizontal white-to-white (WTW) distance and the ACD measured with the scanning-slit corneal topography system following the manufacturer's recommendations.

### 2.3 Surgical technique

All surgeries were performed by the same surgeon (Y.S.). Before surgery, the patients were administered dilating and cycloplegic agents. After peribulbar anesthesia, the pIOL was inserted through a 3.0-mm clear corneal incision with the use of an injector cartridge (STAAR Surgical Co) after the anterior chamber was filled with sodium hyaluronate viscoelastic (PROVISC; Alcon Laboratories, Inc, USA). No preoperative or intraoperative peripheral iridectomies were performed in any case. Centration was ensured before pupillar constriction caused by acetylcholine injection into the anterior chamber. Remaining viscoelastic was removed with gentle irrigation and aspiration. After surgery, tobramycin-dexamethasone (Tobradex; Alcon, Fort Worth, Texas, USA) and levofloxacin (Cravit; Santen, Osaka, Japan) medications were prescribed topically 4 times daily for 7 days, the dose being reduced gradually thereafter.

### 2.4 Outcome assessment

Postoperative examinations were scheduled at 1 day, 1 week, and 1, 3, and 6 months. The evaluations included UDVA, CDVA, manifest refraction, tonometry, ECD, slitlamp microscopy, and fundoscopy. The central vault defined as the distance between the pIOL and the crystalline was measured by ultrasound biomicroscopy (UBM). It was performed by the same examiner (Y.W.) using the SW-3200L full-scale 50 MHz digital system (Tianjin Suowei Electronic Technology Co Ltd, China), as described in our previous study.^[1]^

### 2.5 Statistical analysis

Statistical analysis was performed using SPSS software (version 22.0; SPSS, Inc, USA). Descriptive statistics were obtained. Visual acuity data were converted to logMAR values. Normality of data was checked by the Kolmogorov–Smirnov test. The Wilcoxon signed-rank test was used for statistical analysis to compare the preoperative and postoperative refractive and visual outcomes. One-way analysis of variance (ANOVA) was used to evaluate the vault changes over time. Unless otherwise indicated, the results are expressed as mean ± SD, and differences with a *P* value <0.05 were considered statistically significant.

## 3 Results

**Figure 1:**
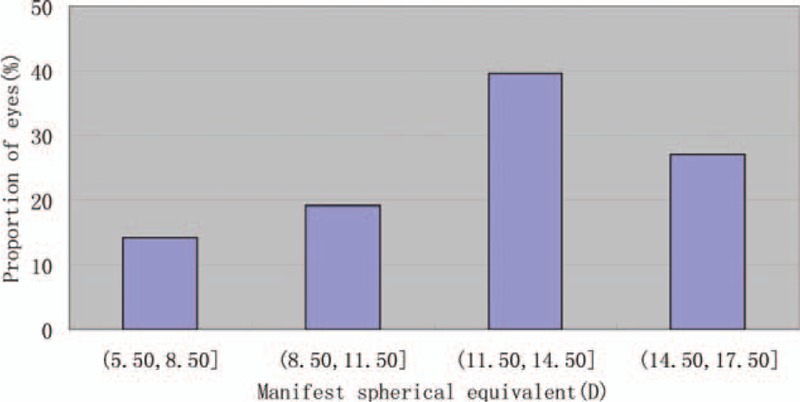
Distribution of manifest SE for the patients preoperatively. SE = spherical equivalent.

**Figure 2:**
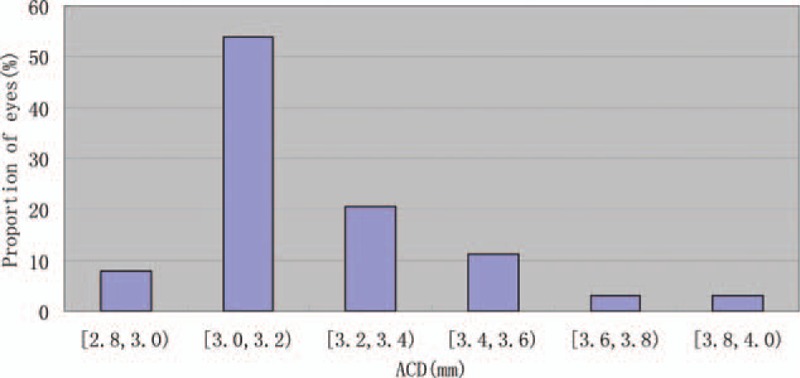
ACD distribution for the patients preoperatively. ACD = anterior chamber depth.

This study enrolled 63 eyes of 32 patients. Table 1 shows the patients’ baseline demographic data and the pIOL characteristics. Figure 1 shows the distribution of the myopic corrections in this study. Figure 2 shows the ACD distribution for the patients. All the patients had uneventful surgery and completed the 6-month follow-up period. Figure 1 shows the V4c pIOL implanted in the eye.

**Table 1:**
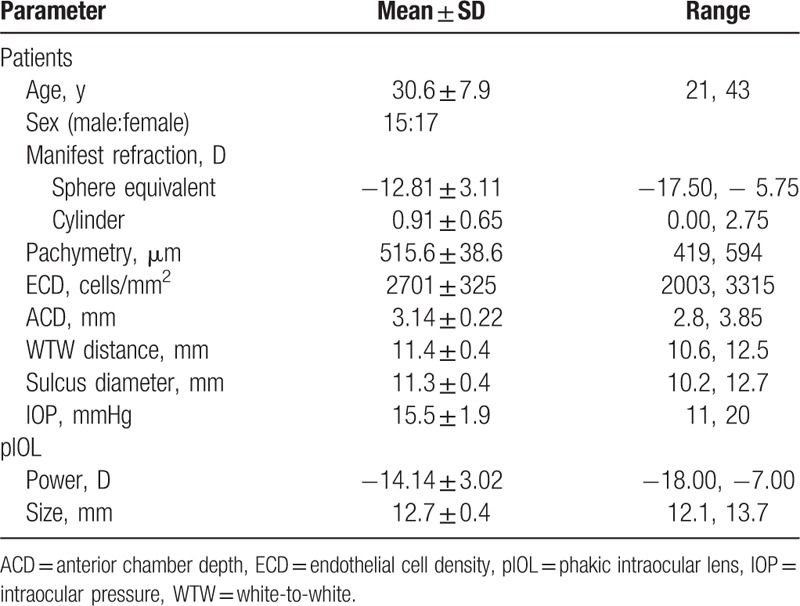
Patients’ baseline demographic data and pIOL characteristics.

### 3.1 Efficacy

The mean postoperative UDVA was 0.134 ± 0.107 logMAR, 0.119 ± 0.098 logMAR, 0.126 ± 0.104 logMAR, and 0.118 ± 0.096 logMAR at 1week, 1 month, 3 months, and 6 months, respectively. Six months after pIOL implantation, the logMAR UDVA was statistically significantly better than the preoperative logMAR CDVA (*P* < 0.001, Wilcoxon signed-rank test). The efficacy index (ratio of postoperative UDVA to preoperative CDVA) was 1.06 ± 0.14, 1.11 ± 0.19, 1.09 ± 0.19, and 1.11 ± 0.19, 1 week and 1, 3, and 6 months after surgery, respectively. A total of 63 eyes (100%) gained ≥2 lines of UDVA, and no eye lost ≥1 line 6 months after ICL V4c implantation. All eyes had a decimal UDVA of 0.5 (20/40) or better at every follow-up visit. Six months after surgery, 77.7% of eyes, and 40.0% of eyes, respectively, had a UDVA of 20/25, and of 20/20 or better.

### 3.2 Safety

**Figure 3:**
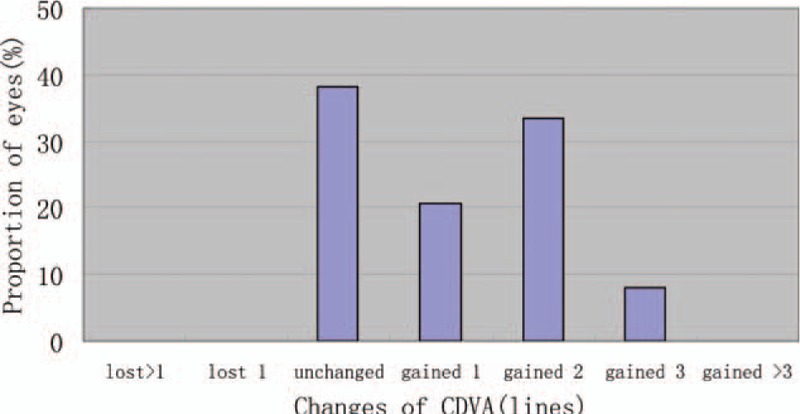
Changes in CDVA from preoperatively to 6 months postoperatively. CDVA = corrected distance visual acuity.

The mean postoperative CDVA was 0.057 ± 0.093 logMAR, 0.036 ± 0.078 logMAR, 0.015 ± 0.048 logMAR, and 0.018 ± 0.035 logMAR at 1 week, 1 month, 3 months, and 6 months, respectively. There was a significant difference between preoperative CDVA and 6-month postoperative CDVA (*P* < 0.001, Wilcoxon signed-rank test). The safety index (ratio of postoperative CDVA to preoperative CDVA) was 1.30 ± 0.34, 1.36 ± 0.32, 1.42 ± 0.30, and 1.42 ± 0.34 at 1 week, and 1, 3, and 6 months after surgery, respectively. A total of 24 eyes (38.1%) had no change in CDVA, 13 eyes (20.6%) gained 1 line, 21 eyes (33.3%) gained 2 lines, 5 eyes (7.9%) gained 3 lines, and no eye lost ≥1 line 6 months after ICL V4c implantation (Fig. 3).

### 3.3 Predictability

**Figure 4:**
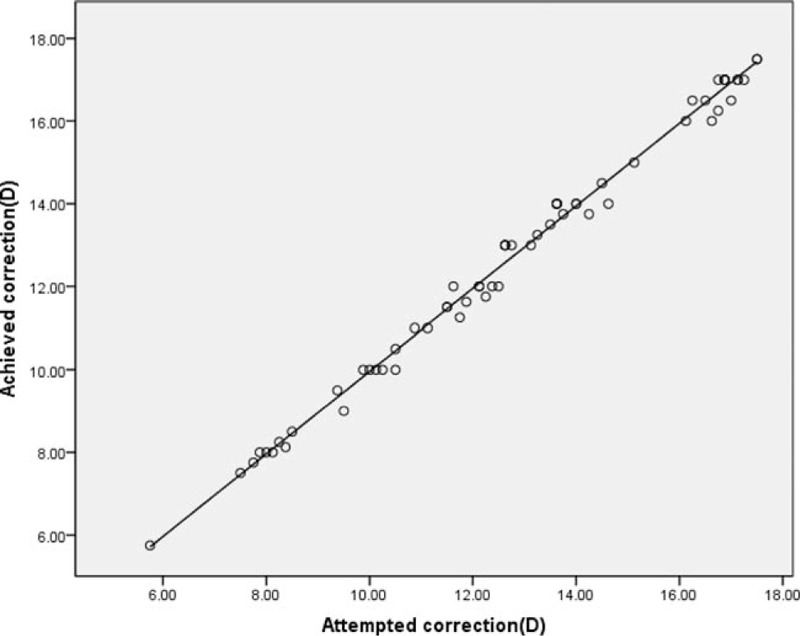
Scatter plot of attempted versus achieved SE correction 6 months postoperatively. SE = spherical equivalent.

Figure 4 shows a scatterplot of the attempted versus the achieved spherical equivalent (SE) correction. Six months after surgery, 61 eyes (96.8%) were within ±0.5 D of the attempted SE, and all eyes (100%) were within ±1.0 D.

### 3.4 Stability

**Figure 5:**
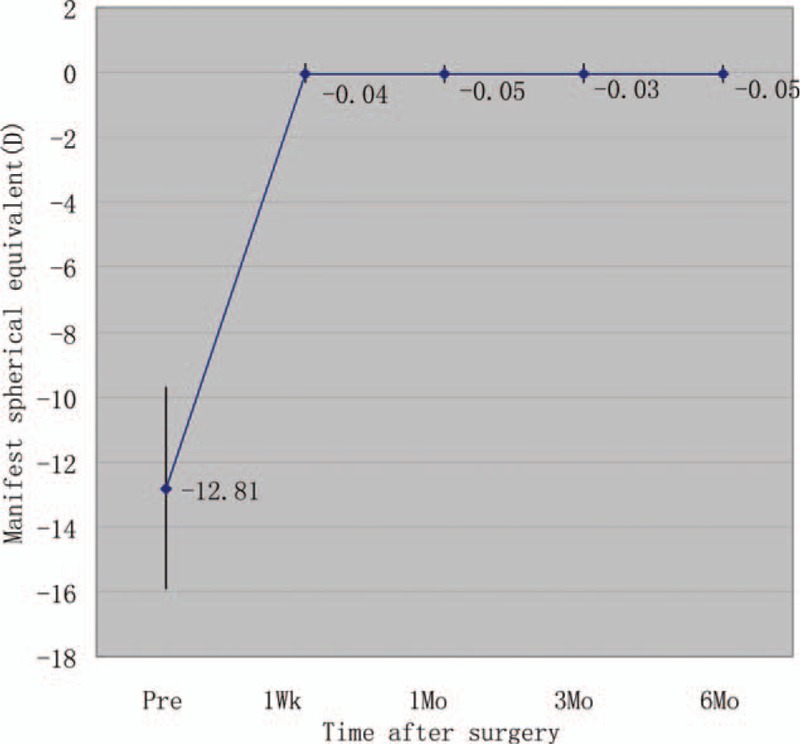
Time course of manifest SE after ICL V4c implantation. ICL = implantable collamer lens, SE = spherical equivalent.

Figure 5 shows the change in the manifest SE and the stability of refraction throughout the follow-up. One week and 1, 3, and 6 months after surgery, the mean manifest SE was −0.04 ± 0.28, −0.05 ± 0.27, −0.03 ± 0.28, and −0.05 ± 0.27 D, respectively. Manifest SE was significantly decreased from −12.81 ± 3.11 D preoperatively to −0.05 ± 0.27 D 6 months postoperatively (*P* < 0.001, Wilcoxon signed-rank test). The change in manifest refraction from 1 week to 6 months was −0.02 ± 0.07 D.

### 3.5 Intraocular pressure

**Figure 6:**
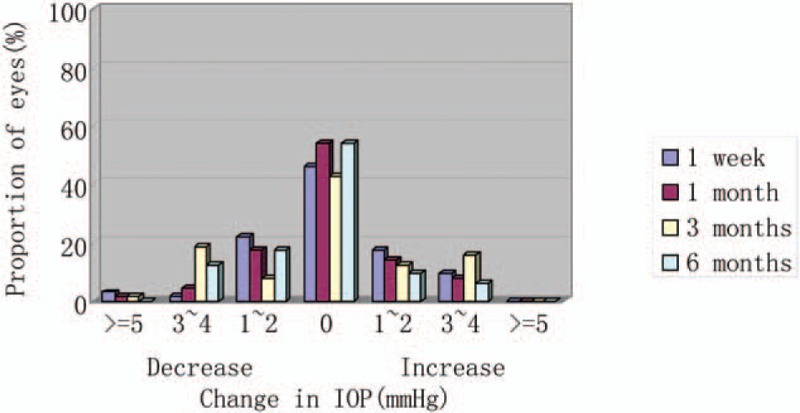
Postoperative changes in IOP. IOP = intraocular pressure.

The mean intraocular pressure (IOP) was 15.5 ± 1.9 mmHg preoperatively. Postoperatively, the mean IOP was 15.6 ± 2.3, 15.5 ± 2.4, 15.4 ± 2.5, and 15.3 ± 2.0 mmHg at 1 week and 1, 3, and 6 months, respectively. No significant increase in IOP (>21 mmHg) occurred in any case during the 6-month follow-up. Figure 6 shows the IOP variations over time. It reflects the percentage of eyes over the whole sample that had an increase or reduction of 1 to 2 mmHg, 3 to 4 mmHg, or >5 mmHg or no variations from baseline to each follow-up visit.

### 3.6 Endothelial cell count

The ECD decreased significantly from 2701 ± 326 cells/mm^2^ preoperatively to 2648 ± 317 cells/mm^2^ 6 months postoperatively (*P* < 0.001, Wilcoxon signed-rank test). The mean endothelial cell loss was 2.0% at 6 months after implantation.

### 3.7 Central vault

The mean central vault measured with UBM was 554.9 ± 287.8 μm (range 160–1080 μm), 513.7 ± 266.4 μm (range 120–1000 μm), and 505.2 ± 258.9 μm (range 120–990 μm) at 1, 3, and 6 months, respectively. There was a trend toward a decrease in central vault over time; however, multiple comparisons showed no significant differences between any 2 periods (*P* = 0.374, one-way ANOVA). Table 2 shows the stratification of central vault with respect to ICL size 6 months after surgery. Pearson correlation coefficient between the central vault and WTW, sulcus diameter, ACD, ICL size was 0.021, 0.119, 0.014, and 0.020, respectively. None of them has significant difference (*P* = 0.869, 0.354, 0.914, and 0.879).

**Table 2:**
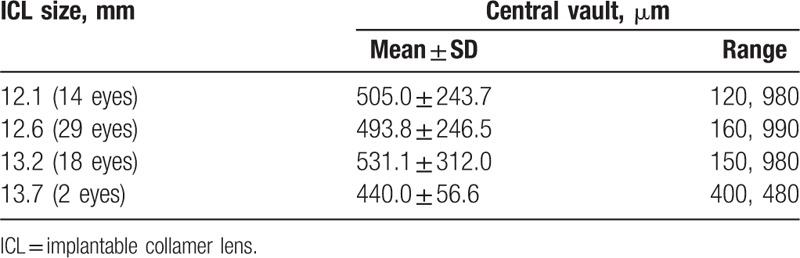
Stratification of central vault with respect to ICL size.

### 3.8 Adverse events and secondary surgeries

There were no perioperative complications, and no eye required pIOL explantation or repositioning. No pigmentary glaucoma, pupillary block, cataract, or other vision-threatening complications occurred during the follow-up.

## 4 Discussion

In the present study, our results of Han Chinese patients with Hole ICL implantation were favorable in all measures of safety, efficacy, predictability, and stability when used for the correction of moderate-to-high myopia, and that no significant IOP rise or cataract formation occurred throughout the 6-month follow-up period, even without preoperative or intraoperative peripheral iridectomy, suggesting its viability as a surgical option for the treatment of such eyes. The new V4c lenses performed well in Han Chinese eyes for the treatment of moderate-to-high myopia. The clinical outcomes were in line with previous studies.^[2,7,10,11]^

With regard to predictability and stability, we obtained stable and predictable refractive outcomes, which were in line with those of previous studies.^[2,7,10]^ The first study of Hole ICL performed by Shimizu et al^[10]^ in 20 myopic eyes (mean SE −7.36 ± 2.13 D) reported 95% and 100% of eyes being within ±0.50 D and ±1.00 D, respectively, of the target correction. Change in manifest refraction from week 1 to month 6 was 0.06 ± 0.28 D. With regard to safety and efficacy, our study had favorable visual acuity outcomes, similar to those reported in previous studies.^[2,10,11]^ Carlos et al^[2]^ evaluated 147 myopic eyes (mean SE −8.80 ± 2.60 D) of 80 patients and obtained favorable UDVA (0.028 ± 0.055 logMAR) and CDVA (0.003 ± 0.013 logMAR) with high safety (1.04) and efficacy (1.00) indices 12 months after pIOL implantation. In Shimizu et al's study,^[10]^ the mean postoperative UDVA and CDVA were −0.25 ± 0.06 logMAR and −0.20 ± 0.09 logMAR, respectively, resulting in a safety index of 1.03 and efficacy index of 1.13 at 6 months. Although all these studies evaluated the same pIOL model, a different amount of myopia may cause slight variations in the outcomes between studies.^[2]^

Despite these good results, there are still concerns about whether the presence of an artificial hole in the center of the optic will deteriorate the optical quality of the V4c Visian by, for example, introducing halos or glare and therefore decreasing the patient's visual performance. However, previous studies^[2,4,11–15]^ concluded that the Hole ICL provided excellent optical quality that was essentially equivalent to that of nonhole conventional ICL. An animal model study by Shiratani et al^[16]^ has reported good and comparable optical quality outcomes of a pIOL with and without a central hole. In the in vitro study by Uozato et al,^[4]^ small differences in the optical performance with negligible clinical effect were found with a pIOL with a 0.36 mm central hole and a conventional pIOL. Higueras-Esteban et al^[6]^ found the complaints about halos have been transient in these patients, suggesting an adaptation process to the presence of the ICL. These results agreed with those reported in the study by Huseynova et al,^[17]^ in which no significant difference between groups, with and without a central artificial hole was found. We are now conducting another study to make a comparison of the optical performance of Hole ICLs and conventional ICLs in Han Chinese patients. So far, the results we have obtained suggest that the central hole in the V4c Visian pIOL does not affect the optical quality and therefore the patient's visual quality. However, more prolonged evaluation with a larger sample of patients is required.

On the other hand, one main concern about the PC pIOL is increased IOP,^[18–22]^ which is associated primarily with pupillary block^[18]^ or with chronic pigment dispersion.^[23]^ In order to prevent the occurrence of the pupillary block, conventional pIOL implantation inevitably requires a preoperative neodymium:YAG iridotomy or intraoperative peripheral iridectomy. In some cases, these complementary procedures can cause discomfort for the patient or intraoperative surgical difficulties.^[18,19,23]^ Hole ICL offers many advantages over conventional ICL, because it eliminates the need for laser iridotomy or iridectomy and, therefore, the potential complications of these additional procedures.^[2]^ Despite these advantages, the port design of the V4c model raises doubt about whether it alone can control postoperative IOP.^[5]^ In the present study, we found no significant variations in IOP (including pupillary block) throughout the 6-month observation period, which agrees with the results of previous studies.^[5–7,10]^ In addition, Higueras-Esteban et al^[6]^ found comparable IOP values 3 months after model V4b and V4c pIOL implantation, even without performing preoperative or intraoperative peripheral iridotomies or iridectomies. Thus, the central hole in the new ICL V4c seems to maintain normal aqueous flow with stable IOP during the follow-up period.

Regarding adverse events, another major concern about ICL implantation is cataract formation.^[6,13,24–27]^ Although the pathogenesis of cataract development, except for surgical trauma, has not been fully elucidated, it is thought to involve direct physical contact between the pIOL and the crystalline lens or malnutrition of the lens resulting from poor circulation of the aqueous humor.^[1,28]^ Several studies^[16,29]^ suggest that the presence of the central hole may contribute to the improvement of the circulation of the aqueous humour to the anterior surface of the crystalline lens and therefore prevents cataract formation. We found no cataract formation in any case throughout the 6-month follow-up, which agrees with findings reported in previous studies.^[2,10–11]^ Furthermore, low pIOL vault was thought to be the most important factor to cause cataract.^[30]^ Long-term observation showed that a minimum central vault of 230 mm is necessary to ensure total clearance of the ICL.^[31]^ Choi et al^[32]^ describe the ideal pIOL vault as between 250 mm and 750 mm. To demonstrate the safety of the new ICL design, it was important to determine whether vault values were comparable with the classical ICL model. The study by Higueras-Esteban et al^[6]^ found there were no significant differences between the ICLs with and without a central hole, which led us to believe that both lenses have a similar anatomic interaction with the intraocular structures. In our study, the mean central vault was 505.2 ± 258.9 μm at 6 months, much greater than these recommended values. This might explain why no lens opacification occurred in our study. However, considering the tendency of vault to decrease over time, the short follow-up is insufficient for detecting such complications. In our previous study,^[1]^ we evaluated longitudinal changes in vault after pIOL implantation. The largest change occurred between 1 month and 3 months, with slight vault change beyond this period. Similarly, this present study showed a trend toward a decrease in vault over time, although multiple comparisons showed no statistically significant differences between any 2 periods. Longer follow-up with more patients is still required to assess long-term safety of the new ICL design.

Endothelial cell loss after Hole ICL implantation has also been evaluated in our study. We found the mean percentage of endothelial cell loss was 2.0% at 6 months after implantation. Some discrepancies exist in the data of endothelial damage reported in previous studies.^[33–36]^ In the study by Alfonso et al,^[7]^ the rate of postoperative endothelial cell loss was ∼8.5% 6 months after pIOL implantation. In another study, Kamiya and coworkers^[10]^ reported a mean endothelial cell loss of 2.8% at 6 months. However, more prolonged observation is necessary to determine the tendency of this cell loss over time.

## 5 Conclusions

In summary, the outcomes in the present study indicate that Hole ICL implantation is safe and effective, and provides predictable and stable refractive results in the correction of moderate-to-high myopia in Han Chinese patients. The central port simplifies surgery and appears to reduce postoperative complications. Long-term evaluation with a larger sample of patients is required to assess the safety and stability of this new design, particularly in terms of the pIOL vault and increased IOP.
